# An empirical comparison of the harmful effects for randomized controlled trials and non-randomized studies of interventions

**DOI:** 10.3389/fphar.2023.1064567

**Published:** 2023-03-21

**Authors:** Minhan Dai, Luis Furuya-Kanamori, Asma Syed, Lifeng Lin, Qiang Wang

**Affiliations:** ^1^ Mental Health Center, West China Hospital of Sichuan University, Chengdu, China; ^2^ School of Public Health, Faculty of Medicine, The University of Queensland, Herston, QL, Australia; ^3^ Department of Population Medicine, College of Medicine, Qatar University, Doha, Qatar; ^4^ Department of Epidemiology and Biostatistics, University of Arizona, Tucson, AZ, United States

**Keywords:** randomized controlled trial, non-randomized studies of intervention, adverse events, harmful effect, empirical comparison

## Abstract

**Introduction:** Randomized controlled trials (RCTs) are the gold standard to evaluate the efficacy of interventions (e.g., drugs and vaccines), yet the sample size of RCTs is often limited for safety assessment. Non-randomized studies of interventions (NRSIs) had been proposed as an important alternative source for safety assessment. In this study, we aimed to investigate whether there is any difference between RCTs and NRSIs in the evaluation of adverse events.

**Methods:** We used the dataset of systematic reviews with at least one meta-analysis including both RCTs and NRSIs and collected the 2 × 2 table information (i.e., numbers of cases and sample sizes in intervention and control groups) of each study in the meta-analysis. We matched RCTs and NRSIs by their sample sizes (ratio: 0.85/1 to 1/0.85) within a meta-analysis. We estimated the ratio of the odds ratios (RORs) of an NRSI against an RCT in each pair and used the inverse variance as the weight to combine the natural logarithm of ROR (lnROR).

**Results:** We included systematic reviews with 178 meta analyses, from which we confirmed 119 pairs of RCTs and NRSIs. The pooled ROR of NRSIs compared to that of RCTs was estimated to be 0.96 (95% confidence interval: 0.87 and 1.07). Similar results were obtained with different sample size subgroups and treatment subgroups. With the increase in sample size, the difference in ROR between RCTs and NRSIs decreased, although not significantly.

**Discussion:** There was no substantial difference in the effects between RCTs and NRSIs in safety assessment when they have similar sample sizes. Evidence from NRSIs might be considered a supplement to RCTs for safety assessment.

## 1 Introduction

Randomized controlled trials (RCTs) are considered the most unbiased study design and represent the current gold standard for assessment of efficacy of interventions (Guyatt et al., 2008). Through the randomization process, RCTs would mostly avoid the bias of confounding factors by indicating the intervention effect (Shrier et al., 2007). However, RCTs are expensive, and thus most RCTs only cover a small number of patients with a short follow-up period (Van Spall et al., 2007; Kennedy-Martin et al., 2015). In addition, sample size estimates for RCTs are usually based on the main outcome, that is, efficacy, rather than adverse events. This makes it challenging to assess safety outcomes since many outcomes occur at a low frequency—the observed events would be rare and even zero for certain outcomes. Therefore, statistical inference faces significant uncertainty caused by random errors (Bhaumik et al., 2012; Efthimiou, 2018). In addition, recruiting subjects usually involves strict inclusion criteria, and researchers tend to exclude high-risk patients, such as children, elderly people, pregnant women, patients with multiple complications, and those with potential drug interactions. These restrictions limit the representativeness of the findings of RCTs (Chou and Helfand, 2005; Golder et al., 2011).

Non-randomized studies of interventions (NRSIs) are an alternative to overcome the aforementioned issues for assessing safety. It is widely known that a case-control study is designed for when the cases of events are rare (Vandenbroucke and Pearce, 2012). There are two sources of error that could impact the estimates of NRSIs, namely, systematic error (bias) and random error. For effectiveness of intervention, the bias of NRSIs is deemed to be the main effect modifier on the results, and the random error may have limited impacts due to the large sample size and sufficient outcomes (Higgins et al., 2011). Methods such as stratification, matching, and regression analysis have been proposed to address the confounding bias for NRSIs (McNamee, 2005; Austin, 2011). Simulation studies have verified that these methods work well to control the impact of confounders on the effects (Jreich and Sebastien, 2021). However, for rare adverse events, such methods may not be feasible due to the limited number of cases. For example, when the event risk is 1/1000, even for an NRSI with a sample size of 2000, the expected number of cases would only be two, which is insufficient for the aforementioned methods. In such a case, in safety assessment, the random error may have a larger impact than the systematic error (bias), which dominates the results.

One increasingly popular method was to pool all available RCTs of the same topic together, i.e., *via* a meta-analysis, to increase the statistical power, and it has the ability to increase the power in testing whether the true effect actually exists. Nevertheless, the statistical power of these meta-analyses was still seriously insufficient (Jia et al., 2021). Researchers then proposed to include NRSIs in the meta-analysis because, for safety outcomes, the primary aim is to capture any signal of harm (Reeves et al., 2013; Valentine and Thompson, 2013). This is somewhat reasonable as we mentioned previously that for safety outcomes of rare events, systematic error may have a limited impact on the results. Even so, this has raised wide controversy as the concerns about the confounding bias still exist for NRSIs and will be synthesized into the pooled effect (Benson and Hartz, 2000; Concato et al., 2000; Ioannidis et al., 2001; Abraham et al., 2010; Hemkens et al., 2016; Soni et al., 2019).

To address this concern, we designed an empirical study based on a database of systematic reviews of safety that compared the effects of RCTs and NRSIs to see whether there was any difference in the evaluation of adverse events between them.

## 2 Materials and methods

The current study findings are reported according to the Strengthening the Reporting of Observational studies in Epidemiology (STROBE) checklist for case-control studies (von Elm et al., 2008). A brief description of the study is as follows. First, we searched for the published systematic reviews of safety and screened for those with safety as exclusive outcomes. Then, we checked the eligible systematic review for those including both RCTs and NRSIs in the meta-analyses. The RCTs and NRSIs were further matched by sample size (1:1) within each meta-analysis. Finally, the effects of each pair of RCT and NRSI were compared.

### 2.1 Sample size estimation

To ensure a sufficient sample size (pairs) for the statistical test, we used the following formula to estimate the minimum sample size for the current study: $n={\left(z_{\alpha /2}\times d/E\right)}^{2}$ (Donner, 1984). Here, E indicates the margin of error and d represents the expected standard deviation of the difference of the effects (i.e., ln odds ratio, lnOR) across the pairs. For the margin of error, it is a concept similar to the bias in a simulation study, namely, how close the estimated effect is to the true effect (Donner, 1984). For the standard deviation, it is a concept similar to the between-study heterogeneity in a meta-analysis (Pateras et al., 2018). Therefore, we took 25% as the tolerable margin of error and 1 as standard deviation, indicating that there would be substantial-to-large heterogeneity across pairs (Ju et al., 2020; Xu et al., 2021a). Based on these parameters, the estimated sample size of the current study is 96.04; that is, we need at least 97 pairs of RCTs and NRSIs to ensure the statistical power to test whether the difference of the effects across the pairs was significant.

### 2.2 Data source

We used a dataset collected in 2020, which was primarily established to improve the evidence-based practice for safety assessment and has been documented elsewhere (Xu et al., 2021b). The dataset consists of 640 systematic reviews of healthcare interventions published in two time periods (2008–2011 and 2015–2020), with adverse events as exclusive outcomes and at least one meta-analysis. The two different periods were primarily designed for comparing how double-zero studies were dealt with by systematic review authors over time (Xu et al., 2021b). For each time period, a comprehensive literature search was performed to ensure the representativeness of the sample (systematic reviews of safety). A detailed description of the dataset can be found in our previous works (Zorzela et al., 2014; Xu et al., 2021b).

### 2.3 Eligibility criteria

We screened 640 systematic reviews for those with at least one outcome (each outcome referred to a separate meta-analysis) that included both RCTs and NRSIs in order to compare the effects of NRSI *vs.* RCT. In addition, considering that data extraction error is commonly seen in published meta-analyses, we only considered those providing summarized 2 x 2 table data for each study in the meta-analysis; a further double-checking process for such data through original studies is possible. Based on the same consideration, those reviews directly reporting the effect size (e.g., OR) and standard error for the meta-analysis were not considered; for such systematic reviews, it is impossible to check whether the effect sizes they used were correctly estimated or extracted, especially for NRSIs. We collected RCTs and NRSIs in systematic reviews under the condition that each pair of the RCT and NRSI has the same topic. Thus, the potential impact of different topics on the results was eliminated. In addition, only pairwise meta-analyses were considered to ensure the interventions were homogeneous.

### 2.4 Data collection

The meta-analytic data of each outcome from each eligible systematic review were extracted by two review authors independently. Any disagreements were solved by discussing with the lead author. These include the 2 x 2 table information (i.e., numbers of cases and sample sizes in intervention and control groups) of each study in the meta-analysis, type of design of each study (i.e., RCT or NRSI), first author of the systematic reviews, and first author and year of publication of included studies. During data extraction, any disagreements were solved by discussion. The primary data were collected from the systematic reviews, and to ensure the quality of the data, we further double-checked the data of matched pairs from the original studies included in the corresponding systematic reviews.

### 2.5 Data analysis

Previous studies pooled the effects of NRSIs and RCTs by treating them as subgroups in a meta-analysis and compared the pooled effects across each meta-analysis (Mathes et al., 2021). However, this method has a big disadvantage in that it requires a sufficient number of studies (i.e., 10) in each subgroup to ensure the robustness of the pooled effects. Under such a limitation, there would be very few meta-analyses that would meet the requirement and may further impact the generalizability of the findings.

In the current study, in order to compare the potential difference of the effects, we matched RCTs and NRSIs within the same meta-analysis by their sample sizes to control the impact of random error on the effects. In brief, we first calculated the sample size of each study in each meta-analysis and ranked the sample sizes within the meta-analysis. Then, those RCTs and NRSIs with similar sample sizes were matched as a pair, using the “nearest neighbor matching” method (Austin, 2011). To ensure the matched RCT and NRIS have almost the same sample size, we calculated the ratio of their sample size; only those with a ratio from 0.85/1 to 1/0.85 were considered to avoid the potential influence of sample size on the results (Xu et al., 2021c).

In each pair, the OR and its standard error of the RCTs and NRSIs were estimated as it has been considered one of the optimal effect estimators (Doi et al., 2020; Doi et al., 2021). For those studies with zero events in single or double groups, the continuity correction was applied by adding 0.5 to each cell to produce an approximate evaluation of the OR and its standard error (Xu et al., 2021d). Furthermore, the ratio of the ORs (ROR) of NRSI against RCT was calculated to reflect the deviation of the effects; the ROR is the primary outcome of the current study (Dechartres et al., 2018). This statistics allows us to further test whether there is a difference in the effect of RCTs and NRSIs. When the weighted mean value of the ROR across the pairs is 1, there would be no difference between the effect of RCTs and NRSIs. In order to obtain the weighted mean value of the ROR, we calculated the natural logarithm of ROR (lnROR) and its standard error and then used the inverse variance heterogeneous model to combine these lnRORs (Doi et al., 2015; Doi and Furuya-Kanamori, 2020). The standard error of the lnROR of each pair can be estimated using the SEs for the RCT and NRSI estimates (Golder et al., 2011).

$$SE\left(\ln ROR\right)=\sqrt{{\left(SE{\ln OR}_{rct}\right)}^{2}+{\left(SE{\ln OR}_{nrsi}\right)}^{2}.}$$

The pooled effect is the weighted mean value. A statistical null hypothesis would be then the pooled lnROR = 0. We used the two-sided t -test with the significant level of alpha = 0.05. Sensitivity analysis was employed by cluster robust error meta-regression to consider the potential correlation of lnRORs for the pairs within each systematic review (Xu and Doi, 2018). Further subgroup analysis by the maximum sample size of each pair was employed to see if the potential difference of the effects varies by sample size. The following five groups were prespecified: 1–50, 51–100, 101–200, 201–500, and >501. Statistical analyses were conducted in MetaXL 5.3 software (EpiGear International, Australia) and Stata 14/SE (Stata, College Station, TX).

## 3 Results

### 3.1 Basic characteristics

Of the 640 systematic reviews of adverse events, 87 included both RCTs and NRSIs. We further excluded 12 with the NRSIs only used for incidence of adverse events or did not include both RCTs and NRSIs within a meta-analysis. Of the remaining 75 systematic reviews, 31 were eligible, which had at least one outcome, contained both RCTs and NRSIs, and provided summarized 2 x 2 table data for each study in the meta-analysis (Grootscholten et al., 2008; Sun et al., 2008; Torloni et al., 2009; Touzé et al., 2009; Slobogean et al., 2010; Yaghoobi et al., 2010; Aires et al., 2015; Geng et al., 2015; Ghayoumi et al., 2015; Inokuchi et al., 2015; Wang et al., 2015; Yoon et al., 2015; Zhang and Ma, 2015; Keir et al., 2016; Peng et al., 2016; Vavken et al., 2016; Balasubramanian et al., 2017; Geminiani et al., 2017; Pecorelli et al., 2017; Cheng et al., 2018; Shah et al., 2018; Zhao et al., 2018; Ceresoli et al., 2019; Craveiro et al., 2019; Jiang et al., 2019; Menne et al., 2019; Nagy et al., 2019; Shah et al., 2019; Vaos et al., 2019; Winberg et al., 2019; Yang et al., 2019). The selection process is reported in Supplementary Figure S1, and the characteristics of the included systematic reviews are shown in Supplementary Table S1.

From the 31 systematic reviews, 178 meta-analyses contained both RCTs and NRSIs with a total of 1,404 studies. 119 pairs of RCTs and NRSIs were successfully matched for the analysis (Supplementary Figure S1). In a further analysis of the 238 studies from 119 pairs, we recorded two (0.84%) had data extraction errors, which were further addressed by correcting these errors. The sample size of the current study is bigger than the minimum requirement (*Sample size estimation*). Among these 119 pairs, there were 19 (15.97%) with the sample size ranging from 1 to 50, 41 (34.45%) pairs ranging from 51 to 100, 19 (15.97%) ranging from 101–200, 17 (14.29%) ranging from 201–500, and 23 (19.33%) with the sample size >500.

### 3.2 RCTs *vs.* NRSIs on the effects

**FIGURE 1:**
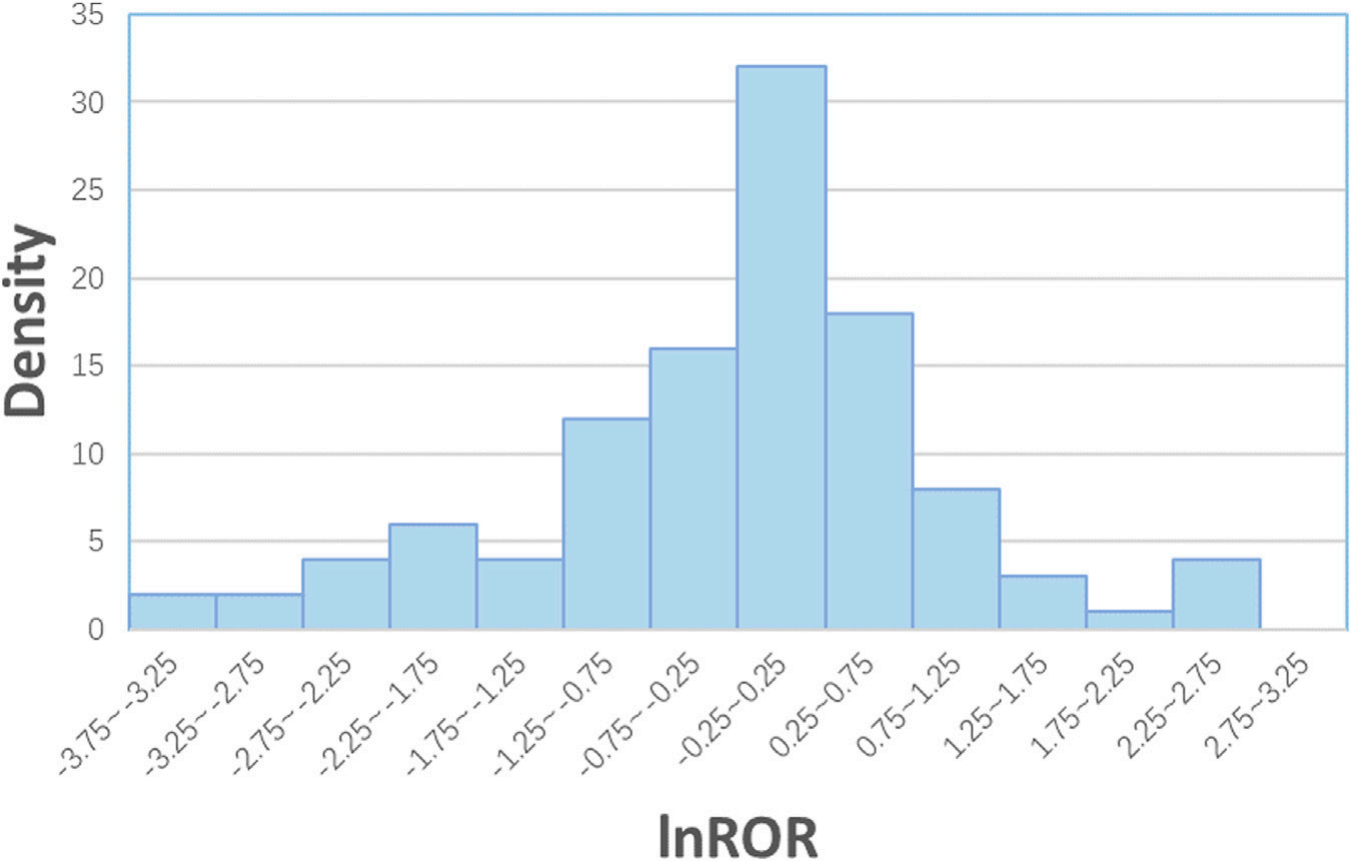
Distribution of lnRORs.

Figure 1 shows the distribution of the lnRORs, which has an approximately normal distribution (*p* = 0.446 for skewness and *p* = 0.13 for kurtosis). The unweighted mean value of the lnROR was − 0.14 with a standard deviation of 1.23, and the single-sample t -test showed no substantial difference of lnROR over zero (t = − 1.25, *p* = 0.21).

Supplementary Figure S2 shows the forest plot of the weighted average lnRORs. Again, no difference was observed between the effects of NRSIs against RCTs. The pooled ROR across the 119 pairs was 0.96 (95% confidence interval [CI]: 0.87, 1.07; *p* = 0.49), with no obvious between-study heterogeneity (I^2^ = 0%). A robust meta-regression model that considers the correlation between the pairs within a systematic review showed a similar result, with the pooled ROR as 0.96 (95% CI: 0.90, 1.03; *p* = 0.27).

### 3.3 Subgroup analysis

**FIGURE 2:**
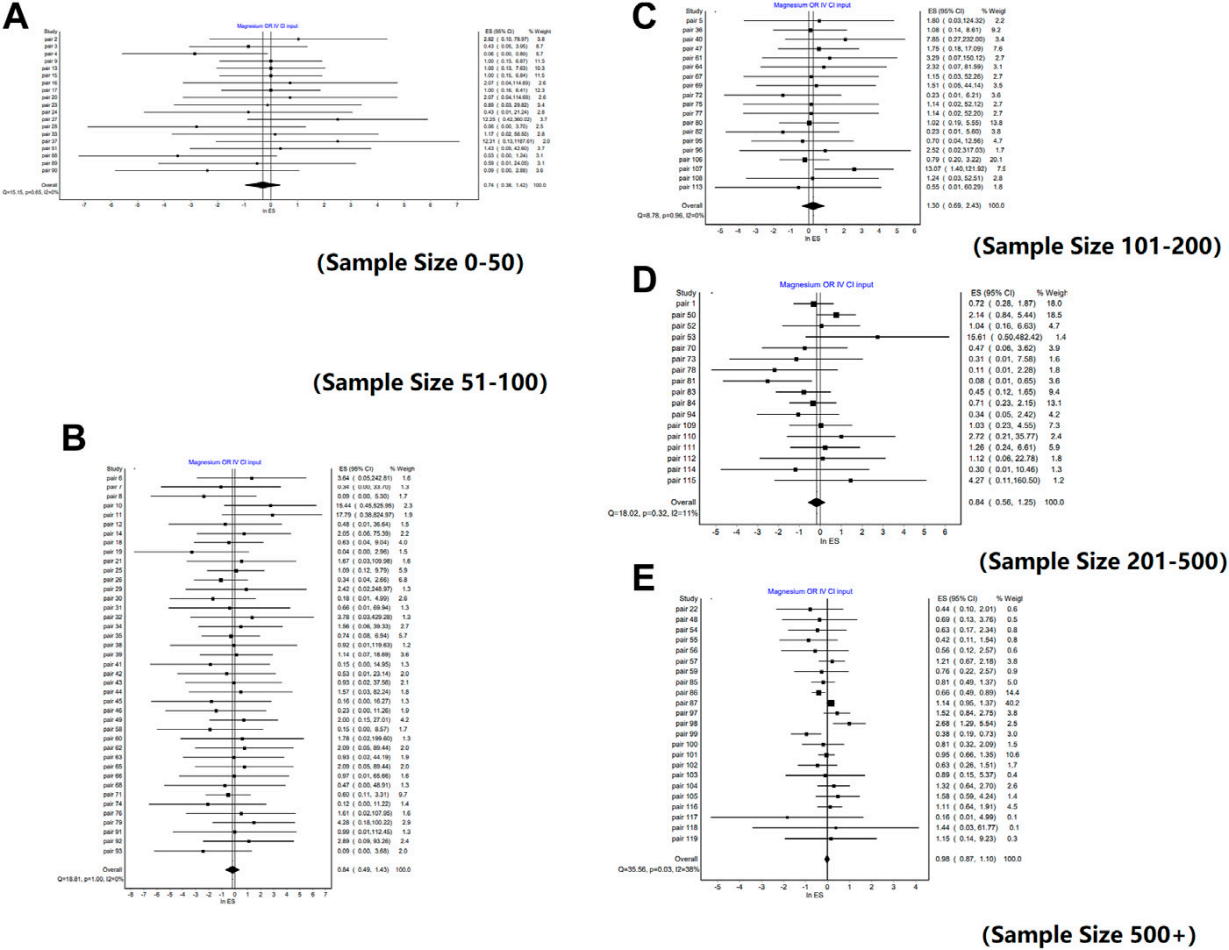
Forest plots of lnROR by sample size. [**(A)** Forest plot of lnROR for pairs with sample sizes between 0 and 50; **(B)** Forest plot of lnROR for pairs with sample sizes between 51 and 100; **(C)** Forest plot of lnROR for pairs with sample sizes between 101 and 200; **(D)** Forest plot of lnROR for pairs with sample sizes between 201 and 500; **(E)** Forest plot of lnROR for pairs with sample sizes, ore than 500].

**FIGURE 3:**
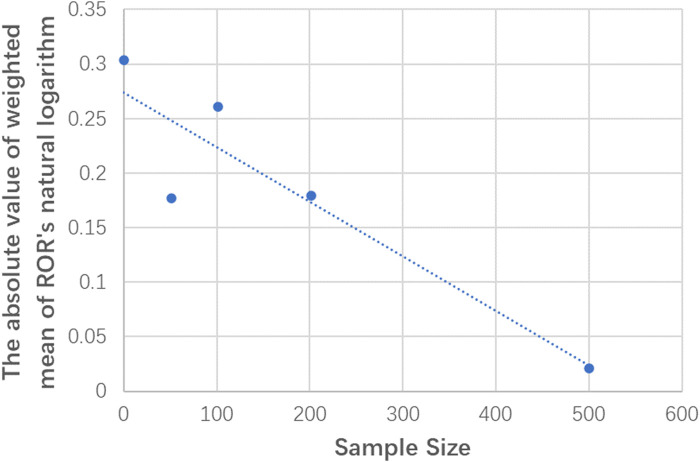
Scatter plot between the sample size and the absolute value of the weighted mean lnRORs.

Similar conclusions were obtained from the analysis of different sample size subgroups. There was no significant difference between the weighted mean value of lnROR and 0 in each subgroup, that is, there was no significant difference in the effects between RCTs and NRSIs, regardless of sample size. The forest plots of the subgroup analyses are shown in Figure 2. However, there was a slight difference in the absolute value of the weighted mean of lnROR for each sample size subgroup, which decreased lnROR with increasing sample size (Figure 3). With the increase in sample size, the difference between RCTs and NRSIs diminished.

In addition, the treatment used in the original study had no significant effect on the results. We compared the weighted mean of lnROR in the treatment subgroup, and the results of either surgical treatment or drug therapy were close to 0, and there was no significant difference (Supplementary Figure S3).

## 4 Discussion

In this study, we compared the effects of RCTs and NRSIs on safety assessment based on empirical evidence. Our results showed that there was no significant difference between RCTs and NRSIs in the evaluation of adverse events of the same topic, and there was no significant difference in sample size or treatment subgroups.

In our research, although different sample size subgroups yielded similar results, there was still a slight difference in the weighted average RORs of different sample size subgroups. As shown in Figure 3, with the increase in the sample size, the value of lnROR decreases gradually; that is, the difference between RCTs and NRSIs gradually decreases. This is likely because the random error decreased as the sample size increased, and the estimated effect is therefore closer to the true effect (i.e., InROR = 0) (Moher et al., 1994; Wang and Ji, 2020). This also indicates that small studies may lead to biased estimation of the effects and should be addressed and interpreted appropriately in further original studies as well as meta-analyses.

Several previous studies have systematically evaluated the differences in the effects of adverse events between RCTs and NRSIs. One study included 19 systematic reviews, and the pooled ROR of RCTs compared to observational studies was estimated to be 1.03 (95% confidence interval 0.93–1.15) (Golder et al., 2011). The other two studies showed similar results (Grodstein et al., 2003; Edwards et al., 2012). These results are similar to our results and further confirm that there is no difference in the average risk estimates of intervention adverse events between RCTs and NRSIs. One possible explanation for the findings is that for safety outcomes, the events are rare, and the sample sizes are also limited, which makes the random error the predominant error impact the effect over the systematic errors (e.g., error from confounding), and therefore under the same sample size with almost the same amount of random error, the effects are similar for RCTs and NRSIs.

However, some minor differences in the effects were observed. A study of postmenopausal hormone therapy on breast cancer survivors found that the results of observational studies were inconsistent with those of randomized trials (Col et al., 2005). This may be due to inconsistencies among the study population that they excluded people with a high incidence of adverse events. In Papanikolaou et al. (2006) study, the authors compared risks of 13 major harms of medical interventions using data from both RCTs and observational studies, and the non-randomized studies were often more conservative in their estimates of risk than the randomized trials. The study attributed these differences to the higher rate of adverse reactions reported by the RCTs because adverse events are recorded more thoroughly in RCTs, owing to regulatory requirements. It may also be caused by the different study populations. Further research on measuring the amount of random error and systematic error on NRSIs for rare events could be useful for the community to better understand the mechanism and deserves more attention.

### 4.1 Strengths and limitations

To the best of our knowledge, our study is currently the largest empirical study that compared the difference of the effects between RCTs and NRSIs for safety outcomes. The sample is representative, and the findings could provide indications for further evidence-based practice for assessing adverse events. In addition, we attempted to source the primary studies contained in each meta-analysis. This can avoid the errors that may exist in the extraction of data by the authors of meta-analyses. Moreover, we matched RCTs or NRSIs with the same outcome in the same systematic review according to their sample sizes, which can avoid the influence of different sample sizes on the results.

The current study has several limitations. First, we did not analyze and evaluate the bias of the included systematic review and possible confounding factors in the original study, such as drug dose, treatment duration, or study population. These confounding factors may affect the outcome of adverse events. In addition, even for the same adverse event, there are differences in how these events were defined or recorded, especially in composite outcomes. The absence of such methodological information increases the potential heterogeneity of the results and even biases the conclusion. Therefore, in the original study, detailed information on outcome collection should be sufficiently provided. Second, selection bias may occur in the current study. It has been estimated that only about 43% of the published studies reported adverse events, while the proportion is 88% in unpublished studies (Golder et al., 2016). This means in the current study, the studies included were those with better reporting on safety outcomes; thus, our results may not be representative of those with poor reporting. Third, we used the matching method for comparison; during the matching process, only 17% were matched among 1,405 studies from the 178 meta-analyses. This means the majority of RCTs and NRSIs have different sample sizes, and therefore whether the effects of them were similar or not is unclear. This is hard to be estimated as the sample size itself is a source of bias. In addition, systematic reviews of adverse events potentially have serious issues in data extraction, and these errors can mislead the conclusions (Xu et al., 2022). Even if data extraction is checked and corrected in this study, there may still be some errors. Further studies are warranted to address these issues.

## 5 Conclusion

In conclusion, the current study identified that there was no significant difference between RCTs and NRSIs in the evaluation of the effect of adverse events for the same topic when they have similar sample sizes. It is of great significance to the systematic reviews of adverse events that well-conducted NRSIs may provide valid results, which is similar to RCTs. Evidence from NRSIs might be considered a supplement to RCTs to improve the generalizability and comprehensiveness of the review.

## Data Availability

The raw data supporting the conclusion of this article will be made available by the authors, without undue reservation.
