# Supplementary material for: An empirical comparison of the harmful effects for randomized controlled trials and non-randomized studies of interventions
Source: Front Pharmacol. 2023 Mar 21;14:1064567. doi: 10.3389/fphar.2023.1064567 (PMC10070801; doi:10.3389/fphar.2023.1064567)
Supplement: Supplementary file 2 [file DataSheet1.docx]

Supplementary Material

# Supplementary Table

**Supplementary Table 1.** Characteristics of included meta-analyses

| Study author | Participants | Intervention | Comparison | Included  pairs  (NRSIs /RCT) | Included participants  per intervention arm  (NRSIs /RCT) | Funding |
| --- | --- | --- | --- | --- | --- | --- |
| Keir 2016 | Neonates | RBC transfusion | No RBC transfusion/Lower RBC transfusion threshold/lower RBC transfusion volumes/RBC transfusions products/Alternative therapy | 2 | 247 vs 254/221 vs 284 | No |
| Nagy 2019 | Juvenile Idiopathic arthritis children | TNF-alpha inhibitor | Placebo/Disease-modifying anti-rheumatic drugs (DMARD) | 2 | 40 vs 40/51 vs 33 | Non-profit |
| Byung-Ho Yoon 2015 | Not restricted | Cemented THA | Cementless THA | 1 | 84 vs 82/75 vs 75 | No |
| Peng 2016 | Not restricted | Hybrid NOTES cholecystectomy (NC) | Classical laparoscopic cholecystectomy (LC) | 3 | 136 vx 146/131 vs 150 | No |
| Yang 2019 | Tic disorders (TDs) | Aripiprazole | Placebo or other types of pharmacotherapies | 13 | 407 vs 328/425 vs 337 | Non-profit |
| Cheng 2018 | Type 2 diabetes mellitus (T2DM) | Rosiglitazone | Other anti-diabetic treatments | 1 | 310 vs 5548/2635 vs 2634 | No |
| Toyama 2015 | After total laryngectomy | Early oral feeding (<5 days) | late oral feeding (>7 days) | 1 | 20 vs 18/21 vs 21 | No |
| Wang 2015 | Patients undergoing permanent colostomy | Extraperitoneal route | Transperitoneal route | 1 | 22 vs 15/18 vs 18 | No |
| Alessandro 2017 | Adult patients requiring sinus floor augmentation surgery | Lateral window conventional approach | Alternative surgical techniques | 2 | 73 vs 50/61 vs 61 | No |
| Vaos 2019 | Complicated acute appendicitis (CAA) in children. | Immediate surgery (IS) | Conservative treatment (CT) | 1 | 19 vs 21/20 vs 20 | No |
| Grootscholten 2008 | Singleton breech pregnancies after 36 weeks of pregnancy | External cephalic version attempt | No external cephalic version attempt was performed | 5 | 183 vs 92/180 vs 98 | No |
| Winberg 2019 | Boys younger than 18 years of age | Mathieu | Tubularized incised plate (TIP) | 8 | 234 vs 360/310 vs 240 | No |
| Geng 2015 | Low anterior resection for rectal carcinoma (LARRC) | Loop ileostomy (LI) | Loop colostomy (LC) | 6 | 372 vs 60/219 vs 225 | No |
| Balasubramanian 2017 | uncomplicated diverticulitis (UD) | Outpatient management | Inpatient management | 1 | 90 vs 46/66 vs 66 | No |
| Menne 2019 | Not restricted | Sodium-glucose cotransporter-2 inhibitors (SGLT2is) | Placebo or active control | 1 | 30 vs 36/34 vs 33 | No |
| Vavken 2016 | Lumbar and cervical spine fusion. | Recombinant human bone morphogenetic protein-2 (rhBMP-2) | Iliac crest bone graft (ICBG) | 4 | 531 vs 503/530 vs 496 | No |
| Shah 2018 | Not restricted | Transcatheter aortic valve implantation (TAVI) | Surgical replacement (SAVR). | 3 | 899 vs 899/883 vs 843 | No |
| Shah 2019 | Not restricted | Transcatheter aortic valve implantation (TAVI) | Surgical replacement (SAVR). | 4 | 1133 vs 1133/1120 vs 1095 | No |
| Ceresoli 2019 | Appendicular stump closure during laparoscopic appendectomy for  acute appendicitis | Endostapler loop ties | Endoscopic loop ties | 3 | 139 vs 137/132 vs 144 | No |
| Inokuchi 2015 | Patients with gastric cancer underwent D1þ  or D2 lymph-node dissection in accordance with the JGCT guide-  lines 2010 | Laparoscopic or laparoscopy-  assisted distal gastrectomy (LDG) | Open distal gastrectomy (ODG) | 7 | 298 vs 320/305 vs 306 | No |
| Pecoreli 2017 | Colorectal surgery | Laparoscopy | Open surgery | 16 | 1712 vs 2049/2146 vs 1513 | No |
| Craveuro 2019 | Not restricted | Statins | No use of statins | 3 | 10623 vs 11525/11116 vs 11088 | No |
| Ghayoumi 2015 | Acute (surgery performed less than 6 weeks from injury) femoral neck fractures in young adults (average age of 50 or younger) | Open reduction with internal fixation (ORIF) | Closed reduction with internal fixation (CRIF) | 3 | 111 vs 6/33 vs 93 | No |
| Slobogean 2010 | Displaced supracondylar fractures of the humerus in children aged between 3 and 12 years | Crossed pin configuration | Lateral pin configuration | 2 | 55 vs 48/52 vs 55 | No |
| Sun 2008 | Adult patients undergoing first-time CABG with or without combined procedures | Aspirin | Aspirin discontinued or placebo given | 4 | 358 vs 255/328 vs 327 | No |
| Torlini 2009 | Low-risk or unselected pregnant women | Exposure to static or B-mode ultrasound alone or associated with continuous or pulsed-wave Doppler or Doppler alone | Not exposure to ultrasonography | 8 | 6776 vs 7106/7062 vs 7183 | No |
| Touze 2009 | Symptomatic and/or asymptomatic stenoses located in the region of the carotid bifurcation | Carotid angioplasty and stenting (CAS) | Carotid endarterectomy (CEA) | 1 | 63 vs 465/127 vs 446 | No |
| Yaghoobi 2010 | Patients with Hp infection and with no evidence of gastroesophageal refl ux disease (GERD) | Helicobacter pylori (Hp) eradication | Persistent helicobacter pylori (Hp) | 2 | 196 vs 99/148 vs 154 | No |
| Zhao 2018 | Patients underwent coronary artery bypass graft (CABG) | Dual antiplatelet therapy (DAPT) | Single antiplatelet therapy | 8 | 990 vs 945/898 vs 885 | Non-profit |
| Zhang 2015 | Human patients older than 18 years undergoing isolated CABG, either on- or off-pump | Preoperative treatment with angiotensin-converting enzyme inhibitors (ACEIs) | Not treatment with angiotensin-converting enzyme inhibitors (ACEIs) | 1 | 3052 vs 3052/3262 vs 2684 | Non-profit |
| Jiang 2019 | Patients suffering allergic rhinitis (AR) with or without asthma (AS) | Allergen-specific immunotherapy (AIT) | Conventional immunotherapy | 3 | 1584 vs 858/1182 vs 1135 | No |

# Supplementary Figure


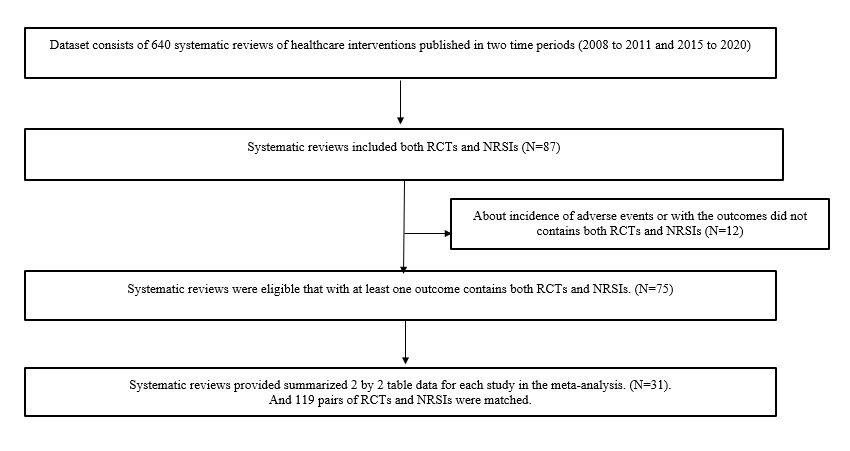


**Supplementary Figure 1.** Flow plot of literature search and screening.


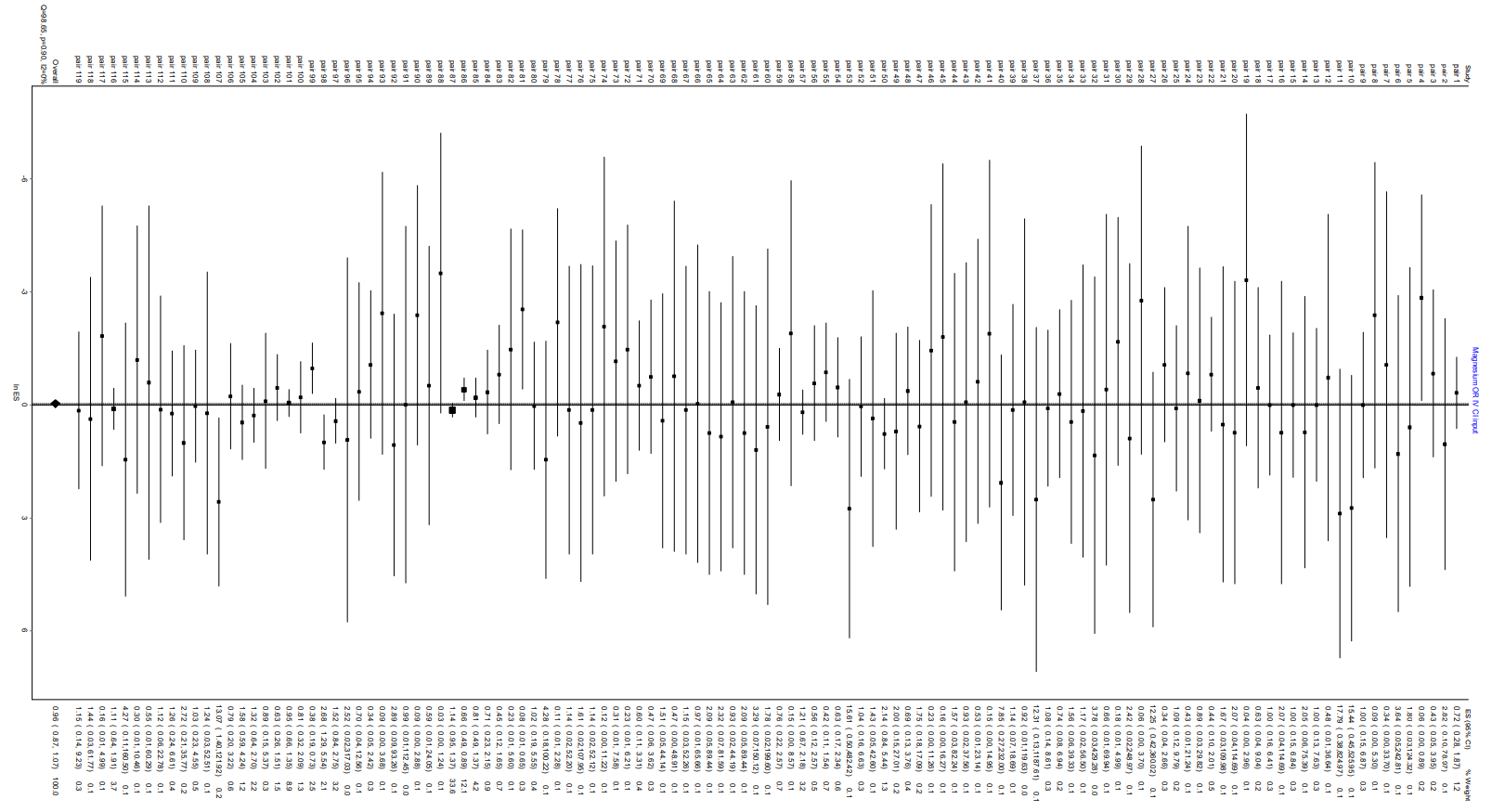


**Supplementary Figure 2.** The forest plot of ROR


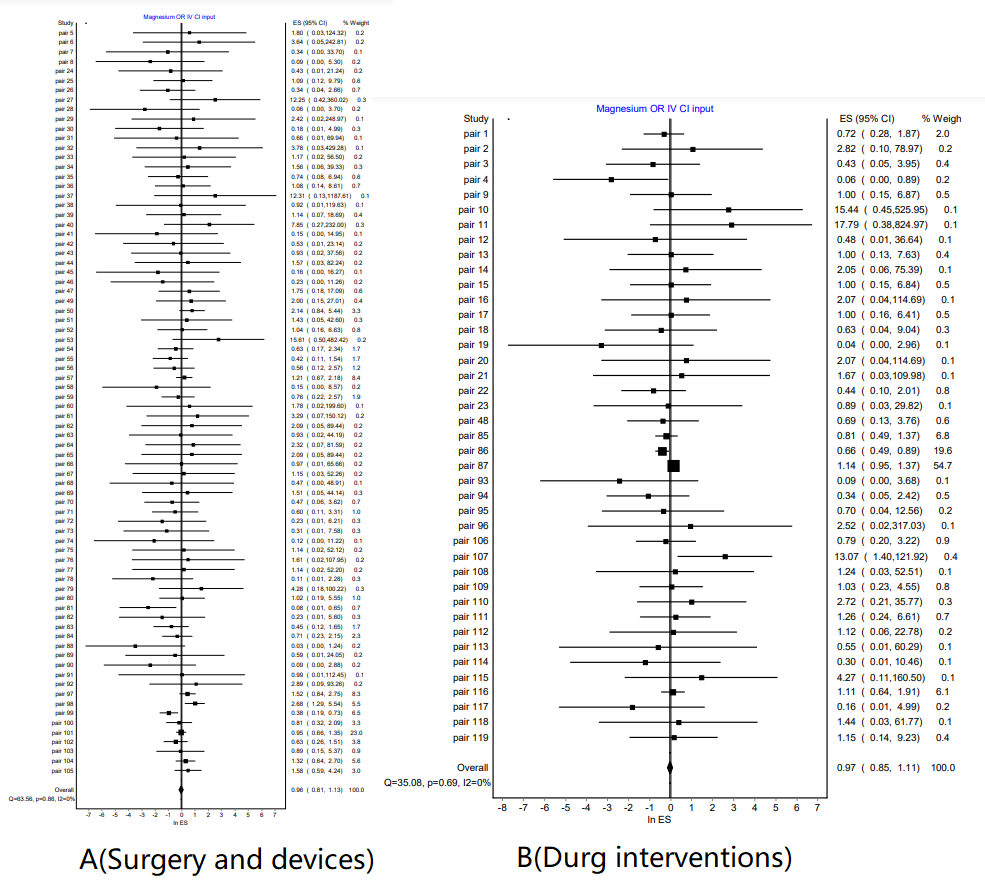


**Supplementary Figure 3.** The forest plots of ROR with different treatment subgroup.
